# Prenatal immune programming of the sex-dependent risk for major depression

**DOI:** 10.1038/tp.2016.91

**Published:** 2016-05-31

**Authors:** S E Gilman, S Cherkerzian, S L Buka, J Hahn, M Hornig, J M Goldstein

**Affiliations:** 1 Health Behavior Branch, Division of Intramural Population Health Research, *Eunice Kennedy Shriver* National Institute of Child Health and Human Development, Rockville, MD, USA; 2 Department of Social and Behavioral Sciences, Harvard T. H. Chan School of Public Health, Boston, MA, USA; 3 Department of Epidemiology, Harvard T. H. Chan School of Public Health, Boston, MA, USA; 4 Department of Psychiatry, Massachusetts General Hospital, Boston, MA, USA; 5 Connors Center for Women’s Health and Gender Biology, Boston, MA, USA; 6 Departments of Psychiatry and Medicine, Harvard Medical School, Boston, MA, USA; 7 Department of Epidemiology, Brown University School of Public Health, Providence, RI, USA; 8 Center for Infection and Immunity, Columbia University Mailman School of Public Health, New York, NY, USA; 9 Department of Epidemiology, Columbia University Mailman School of Public Health, New York, NY, USA; 10 Division of Psychiatric Neuroscience, Massachusetts General Hospital, Boston, MA, USA

## Abstract

Maternal immune functioning during pregnancy contributes to sex-dependent deficits in neurodevelopment and to behaviors associated with affective traits in preclinical studies, and has been indirectly associated with offspring depression in epidemiologic studies. We therefore investigated the association between immune activity during pregnancy and the risk of depression among male and female offspring. We conducted a case–control study of depression (*n*=484 cases and *n*=774 controls) using data from the New England Family Study, a pregnancy cohort enrolled between 1959 and 1966 that assessed psychiatric outcomes in adult offspring (mean age=39.7 years). We assayed concentrations of three pro-inflammatory cytokines, interleukin (IL)-1β, IL-6 and tumor necrosis factor (TNF)-α, and the anti-inflammatory cytokine, IL-10, in maternal serum collected at the end of the second and beginning of the third trimesters. High maternal TNF-α was associated with reduced odds of depression among both male and female offspring (odds ratio (OR)=0.68; confidence interval (CI)=0.48, 0.98). However, when considering the TNF-α to IL-10 ratio, a measure of the ratio of pro- to anti-inflammatory loading, maternal immune effects on offspring depression differed significantly by sex (*χ*^2^=13.9, degrees of freedom=4, *P*=0.008). Among females, higher maternal TNF-α:IL-10 was associated with reduced odds of depression (OR=0.51; CI=0.32, 0.81), whereas, among males, high maternal TNF-α:IL-10 was associated with elevated odds of depression (OR=1.86; CI=1.02, 3.39). Thus, the balance between TNF-α and IL-10 in maternal prenatal serum was associated with depression in a sex-dependent manner. These findings are consistent with the role of TNF-α in the maturation of the sexually dimorphic fetal brain circuitry that regulates stress and affective responses, and support a prenatal stress-immune model of depression pathogenesis.

## Introduction

Major depressive disorder (MDD) is a leading cause of disability, given its high prevalence, early onset, persistence in adulthood and impact on social and economic functioning. Depression also disproportionately affects females.^1^ As with other severe forms of psychiatric illness, evidence suggests that prenatal life is a sensitive period for the establishment of lifelong risk for depression. Such evidence is consistent with a prenatal stress model in which excess maternal glucocorticoids triggered by prenatal stress can alter the fetal stress response circuitry, conferring long-term psychiatric vulnerability.^2^ The current study investigates the role of maternal prenatal immune activity, which is strongly linked with prenatal stress (that is, excess maternal glucocorticoid release), in the risk of depression in male and female offspring in adulthood.

Ecologic studies suggest a possible link between maternal immune disruption and offspring depression. Women exposed to the 1957 A2 influenza epidemic during the second trimester of pregnancy gave birth to children at increased risk for depression as adults.^3^ In contrast, a prospective study of clinically documented prenatal infection did not show associations with subsequent depression.^4^

The strongest evidence to date comes from preclinical studies using experimental *in utero* maternal immune challenges (both viral- and bacterial-type) to induce increased pro-inflammatory cytokines and thereby activate the maternal hypothalamic–pituitary–adrenal (HPA) axis, stimulating glucocorticoid release.^5, 6^ Such immune challenges can elicit long-lasting depressive-like behaviors in rodent offspring.^6, 7, 8, 9^ Studies that investigated effects according to offspring sex found pronounced differences, but not in consistent directions. In some studies, effects were larger in females than in males^10^ (for example, females released more corticosterone following gestational immune challenge),^11^ whereas in other studies males were disproportionately at greater risk (for example, in the domains of neurodevelopmental and behavioral deficits).^6, 11, 12^ The broader inference from these studies is that elevation of maternal pro-inflammatory cytokines affects fetal brain development and alters the fetal innate immune system in a sex-dependent manner.^13, 14^

Preclinical studies investigating the neurobehavioral consequences of gestational immune stimulation have primarily focused on second and third trimesters of pregnancy, a critical period for the organizational effects of gonadal hormones on sexually differentiated brain development.^15, 16^ Thus, offspring sex is critical for understanding the impact that prenatal maternal immune disruptions during this gestational period may have with respect to sex-dependent risk for depression.^2^

Although evidence suggests the importance of maternal immune activation during pregnancy for risk of depression in the offspring, there has been no direct test of this hypothesis in humans. Accordingly, we investigated the association between biomarkers of maternal immune activity in serum drawn at the end of the second and beginning of the third trimesters and the risk of MDD in adult offspring using data from the New England Family Study (NEFS).^17, 18^ We measured concentrations of three pro-inflammatory cytokines—interleukin (IL)-1β, IL-6 and tumor necrosis factor (TNF-α)—and one anti-inflammatory cytokine, IL-10, in maternal serum. (Although IL-6 can be anti-inflammatory, its impact has been considered pro-inflammatory in many contexts,^19^ and it is thus treated as a pro-inflammatory agent here, as in many other studies.) TNF-α, IL-1β and IL-6 were selected for assay as they are the major co-activators of HPA axis function^20^ and thus serve as important indicators of signaling along stress-immune pathways. Further, the receptors for IL-1β, IL-6 and TNF-α are located in brain regions implicated in the stress response circuitry such as the hippocampus and ventromedial and paraventricular hypothalamic nuclei.^21^ These regions are also implicated in depression and are highly sexually dimorphic at the volume level in humans^22^ and nuclei level in animals.^16^ IL-10 was chosen, given its anti-inflammatory property, and is important for understanding the mother’s ability to balance pro-inflammatory with anti-inflammatory responses.^20^ We hypothesized that higher concentrations of maternal pro-inflammatory cytokines (and lower concentrations of the anti-inflammatory cytokine IL-10) would be associated with an elevated risk of major depression in offspring. We further hypothesized that the association between concentrations of pro-inflammatory cytokines in maternal serum and offspring depression would be larger among female than male offspring.

## Materials and methods

### Ascertainment of cases and controls

The NEFS comprises a series of follow-up studies of adult offspring born to mothers enrolled in the New England (Massachusetts and Rhode Island) cohorts of the Collaborative Perinatal Project (CPP). These two cohorts enrolled 13 464 women during one or more pregnancies between 1959 and 1966. The CPP protocol included serial collection of maternal serum samples throughout pregnancy (approximately one draw per trimester) and on the day of delivery; samples were stored at NIH repositories at −20 °C.

Depression cases (*n*=484) and controls without depression (*n*=774) were identified among CPP offspring who participated in three previous NEFS follow-up studies.^17, 18, 23^ Informed consent was obtained from all participants. The current investigation was overseen by the Institutional Review Boards of Brigham and Women’s Hospital and Harvard TH Chan School of Public Health.

Cases met Diagnostic and Statistical Manual, fourth edition (DSM-IV), criteria for Major Depressive Episode. Controls without depression were sampled from the same NEFS follow-up studies from which cases were identified, and have comparable distributions of race/ethnicity and age. Depression was assessed using either the Structured Clinical Interview for DSM-IV (SCID) or the Composite International Diagnostic Interview (CIDI). Both are reliable instruments for assessing MDD, and CIDI diagnoses of MDD are highly concordant with SCID diagnoses.^24^ Recurrent cases of depression were identified based on participants’ reports of two or more lifetime episodes (*n*=277). Control criteria excluded subthreshold cases of MDD—either SCID diagnoses of a ‘possible’ depressive episode or depressive episodes recorded by the CIDI with only two to four symptoms (rather than five or more, as required by DSM-IV). Participants with lifetime SCID diagnoses of psychotic and bipolar disorders were excluded from the study. CIDI modules for these diagnoses were not administered, but we excluded CIDI participants who reported ever being hospitalized for bipolar disorder and participants who endorsed either of two cardinal symptoms of psychosis, ‘hearing voices’ or ‘believed people were plotting against you.’

### Prenatal serologic assays

We assayed stored maternal serum drawn from the end of the second to the beginning of the third trimesters, which coincides with sexual differentiation of brain development, for concentrations of the four cytokines.^15, 16^ Previous work, using samples stored under similar conditions and for a similar length of time (>40 years), demonstrated the long-term stability of analytes from CPP samples.^25^ All samples were handled identically, using assay kits and reagents from a single individual lot, and all assays were completed within a 2-month period. Maternal cytokine levels were assessed using a multiplexed, bead-based immunoassay (Milliplex human cytokine panel, MPXHCYTO-60 K, Millipore, St Charles, MO, USA) on a Luminex 3D detection platform (Luminex, Austin, TX, USA). Assay sensitivities for each cytokine, in pg ml^−1^, were as follows: IL-1β, 0.4; IL-6, 0.3; TNF-α, 0.1; IL-8, 0.2; and IL-10, 0.3. Twenty-five microliters of each serum sample was diluted 1:1 in Assay Buffer and run with six serial dilutions (3.2–10 000 pg ml^−1^) of cytokine standards with two quality-control and one normal serum standards on each 96-well plate.^26^ All samples, standards and controls were run in duplicate. To meet the standards of our quality-control protocols, all kit-provided control samples and a Center for Infection and Immunity in-house control sample must fall within the anticipated ranges both within and across plates. Assays were completed according to the manufacturers’ protocols, with overnight incubation at 4 °C on a shaker before detection of the mean fluorescence intensities of analyte-specific immunoassay beads by Luminex 3D. Raw data (mean fluorescence intensities) were captured using the Luminex xPONENT software (Luminex, v.4.0.846.0), and concentrations of immune factors in each sample were interpolated from standard curves using a five-parameter, weighted, logistic regression curve equation in Milliplex Analyst (v.3.5.5.0). Measurements below the lower limit of detection of any analyte were recoded to the mid-point between zero and the limit of detection for that analyte.^27^ For measurements at or above the upper limit of analyte detection, samples were assayed again at multiple serial dilutions using Assay Buffer to bring concentrations into detectable range.

### Statistical analysis

The cytokines were categorized into quintiles for analysis in order to assess potential nonlinearities in the effects of cytokines on offspring depression. Associations between the quintiles of each cytokine in maternal serum and risk of offspring major depression were evaluated using logistic regression. Analyses were controlled for race (white versus non-white), age at adult interview, gestational age when prenatal serum was obtained and diagnostic assessment instrument (CIDI versus SCID). Estimates of variance were adjusted for the presence of 158 siblings (74 pairs, 2 trios and 1 quartet) in the case–control sample using generalized estimating equations.

Separate models were fitted for each cytokine. For cytokines with significant associations with offspring depression, we fitted additional models to evaluate their relative pro-inflammatory effects by analyzing the ratio of the pro-inflammatory cytokine to the level of IL-10. We set as the reference group for each cytokine the quintile associated with the lowest level of inflammation (that is, the lowest quintile for the pro-inflammatory cytokines, IL-1β, IL-6 and TNF-α, and the highest quintile for the anti-inflammatory cytokine, IL-10). Similarly, the lowest quintile was selected as the reference category for each pro-inflammatory cytokine:IL-10 ratio. Using this approach, the odds ratios (ORs) are interpreted as the magnitude at which higher levels of maternal immune activity relates to the offspring’s risk of depression. We tested for sex differences in the association between maternal cytokines and offspring depression by adding sex and sex-by-cytokine quintile interactions to the logistic regressions, from which we obtained ORs for females and males separately.

Primary analyses were conducted among all participants included in the case–control sample. We conducted further analyses that removed participants with only one lifetime episode of depression from the case group, generating a recurrent-MDD case–control sample. These analyses were performed to reduce the heterogeneity among depression cases and are motivated by evidence, suggesting that early-life stress models of depression may be most relevant for recurrent forms of the disorder.^28^ Sensitivity analyses were conducted in which we excluded participants with prenatal serum samples obtained at delivery (*n*=114), instead of earlier in gestation.

## Results

**Figure 1:**
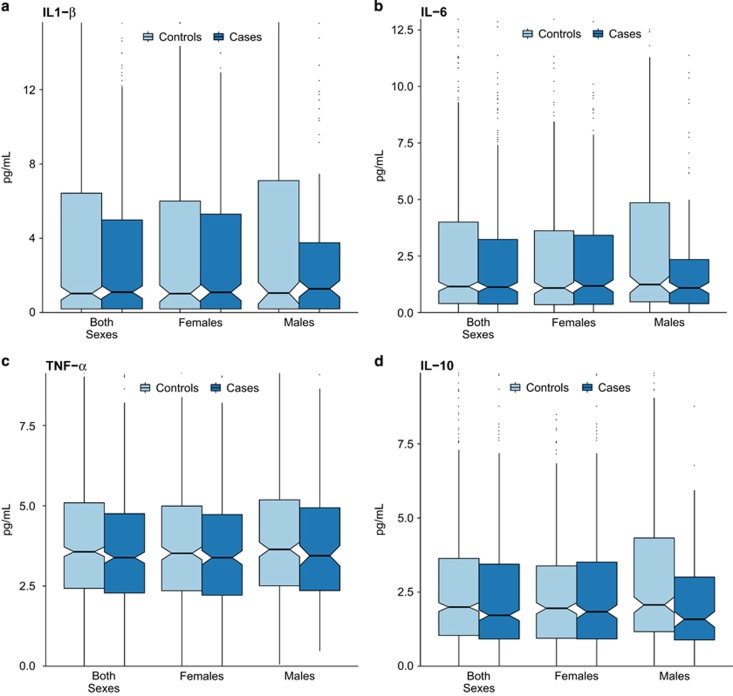
Distributions of IL1-β (**a**), IL-6 (**b**), TNF-α (**c**), and IL-10 (**d**) concentrations in late-second and early-third trimester maternal serum among depression cases and controls without depression (*n*=1258). The *y* axis of each graph extends to the 90th percentile of the distribution of each cytokine. The center lines of each box represent the median, and the diagonal notches in each box represent 95% confidence intervals around the median. IL, interleukin; TNF, tumor necrosis factor.

**Figure 2:**
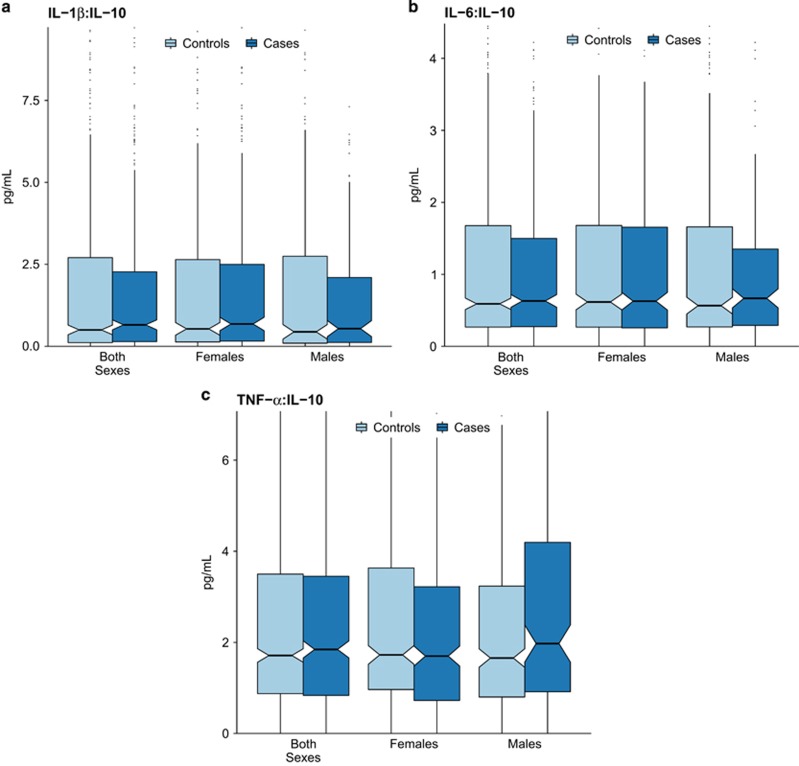
Distributions of pro- versus anti-inflammatory (IL-10) cytokine concentrations (IL-1β:IL-10 in panel **a**; IL-6:IL-10 in panel **b**; TNF-α:IL-10 in panel **c**) in late-second and early-third trimester gestation maternal serum among depression cases and controls without depression (*n*=1258). The *y* axis of each graph extends to the 90th percentile of the distribution of each pro:anti-inflammatory ratio. The center lines of each box represent the median, and the diagonal notches in each box represent 95% confidence intervals around the median. IL, interleukin; TNF, tumor necrosis factor.

There were 484 cases of major depression in the sample (277 of whom had two or more lifetime episodes) and 774 controls without any lifetime history of depression. The demographic characteristics of the sample are shown in Table 1, along with the median concentrations of IL-1β, IL-6, IL-10 and TNF-α in maternal serum. (Median concentrations within each quintile of the cytokines for all cases of depression are shown in Supplementary Table S1, and for recurrent depression cases in Supplementary Table S2.) The mean gestational age at prenatal sample collection was 32.4 weeks (s.d.=3.4 weeks). The mean age of participants’ offspring at the time of adult interview was 39.7 years (s.d.=3.6 years; range 30–50 years). Participant age did not differ by case/control status. Boxplots illustrating the distributions of cytokines between cases and controls, by sex, are presented in Figure 1 (for each cytokine individually) and Figure 2 (for ratios of the pro-inflammatory cytokines to IL-10).

Analyzing male and female offspring together, only the concentration of maternal TNF-α was significantly associated with depression risk in the offspring. Offspring exposed prenatally to *higher* levels of TNF-α (that is, TNF-α concentration in the highest quintile) had a *lower* odds of depression (OR=0.68; confidence interval (CI)=0.48, 0.98; Table 2). We then examined the ratio of maternal TNF-α to IL-10 in relation to offspring depression, and observed a significant sex difference (*χ*^2^ for the sex*TNF-α:IL-10 interaction=13.9; degrees of freedom=4, *P*=0.008), because of opposing associations of the TNF-α:IL-10 ratio with depression for female versus male offspring (see final column, Table 2).

Among female offspring, *in utero* exposure to *higher* TNF-α levels relative to IL-10 was associated with a *lower* risk of depression (OR=0.51; CI=0.32, 0.81). In contrast, among male offspring, exposure to higher maternal TNF-α levels relative to IL-10 was associated with a *higher* risk of depression (OR=1.86; CI=1.02, 3.39). These results remained unchanged when contrasting recurrent cases of depression with non-depressed controls (see Supplementary Table S3), as well as in a sensitivity analysis that removed offspring linked to prenatal serum samples obtained on the day of delivery (*n*=114, Supplementary Table S4).

## Discussion

This study investigated the association between gestational immune activity and offspring’s risk of major depression in adulthood, and examined whether this association differed by sex of the offspring. In doing so, we addressed the hypothesis that alterations in maternal immune activity during pregnancy impair fetal brain development, resulting in long-term sex-dependent vulnerability to the development of depression. Results showed that exposure to higher levels of pro-inflammatory maternal immune activity, specifically a higher concentration of the pro-inflammatory cytokine, TNF-α, in maternal sera relative to the anti-inflammatory cytokine, IL-10, was associated with a *lower* risk of depression among female offspring. In contrast, higher TNF-α:IL-10 was linked to a *higher* risk of depression among males.

Our results suggest that variations in maternal immune system activity during pregnancy, as indicated by circulating maternal cytokines, have sex-dependent effects on the development of major depression in offspring. Previous studies have reported associations between gestational immune biomarkers and other psychiatric conditions, including autism, schizophrenia and bipolar psychoses.^2, 18, 29^ To our knowledge, ours is the first study to relate serologic biomarkers of maternal immune activity to the risk of MDD in offspring.

We speculate based on our results that the abnormal immune system activity frequently observed in adult depression^30, 31^ may originate in part during fetal development. Several studies report marked hypersecretion of pro-inflammatory (Th1) cytokines, including IL-1β, IL-6 and TNF-α, among adults with depression.^32, 33^ However, these studies of adults,^32, 33^ many of which are cross-sectional, are limited in their ability to discern the role of cytokines in the *ontogenesis* of depression, given that the relation between immune markers and depression in adults is likely bidirectional.^34^ In contrast, our study linked *prenatal maternal* immune activity with the offspring’s risk of depression in adulthood. In rodent models, *in utero* viral infection mimicked by the administration of polyriboinosinic:polyribocytidylic acid (poly(I:C) resulted in the post-pubertal emergence of brain immune changes (specifically, IL-1β and TNF-α) and anxiety-related behaviors in the offspring.^35^

*In utero*, indirect markers of maternal immune activation (for example, exposure to an influenza epidemic) have been associated with several neuropsychiatric disorders including depression.^3^ In preclinical studies in which the pregnant dam was exposed to stressful stimuli that induce an inflammatory response marked by increased pro-inflammatory cytokines, the HPA axis was activated, releasing glucocorticoids and HP-gonadal hormones in the mother.^36, 37^ These preclinical studies further demonstrated long-lasting impact on offspring brain development, anxiety behavior and the fetal innate immune system.^5, 35, 38, 39, 40, 41, 42, 43, 44^ The dose of the immunogen, animal species and strain, and sex of the offspring, as well as the gestational timing and acute versus chronic nature of the immune challenge are key factors in the induction of different neurophysiological, behavioral and immunologic responses in the offspring.^5, 6, 8, 37^ For example, a recent preclinical study demonstrated an association of first trimester prenatal stress-induced placental inflammation with deficits in immune-related (IL-6 and IL-1β) gene expression in placental tissue, but only in male offspring.^45^

Findings from experimental injections of viral- or bacterial-type stimuli in pregnant rodents have shown that *increased levels of* pro-inflammatory cytokines during prenatal development were associated with an *increased* risk of depressive-like behavior in adult offspring. Of note, most of these studies were conducted in male animals. Our finding that the depression risk after exposure to higher prenatal TNF-α:IL-10 levels was restricted to male offspring is consistent with this prior work in male animal models.

Our results demonstrating that *higher* pro-inflammatory:anti-inflammatory drive (TNF-α:IL-10 ratio) in maternal serum was associated with a *lower* risk for depression in female offspring were unexpected. However, similar findings have been reported in two prior ecologic studies of prenatal influenza exposure on affective disorders in offspring and in our prior study of sex-dependent risk of psychosis in the NEFS.^18, 46, 47^ Brown *et al.* and Takei *et al.* found that gestational exposure to influenza was associated with a lower risk of affective psychosis in women.^46, 47^

TNF-α, IL-1β and IL-6 are the primary activators of the HPA system,^20^ and glucocorticoids inhibit all three cytokines. In fact, there is a ‘hierarchy of sensitivity,’ with TNF-α being *the most* sensitive to such inhibition at the physiologic level, IL-1β being second and IL-6 being the most resistant.^20^ Thus, mothers undergoing stressful conditions (for example, obstetric complications, trauma, chronic social stress and certain infections) would be expected to produce excess maternal glucocorticoids that would *inhibit* TNF-α responses (that is, the most physiologically sensitive cytokine to HPA axis activation^20^). In fact, prior work suggests that these prenatal HPA axis disruptions are likely to have a greater impact on brain circuitry in female than in male offspring.^2, 16^

TNF-α has both neuroprotective and neurotoxic effects on brain development and brain function.^48^ It is regulated by several hormonal and metabolic factors specific to pregnancy. Pregnancy itself represents a different endocrine and inflammatory state than non-pregnancy, given the necessity to create an environment that will not reject the fetus.^49^ For example, at levels typical of pregnancy, high-dose estradiol treatment of adult female mice reduced Th1 cytokines (including TNF-α), and shifted the balance toward a Th2 anti-inflammatory state,^50^ in contrast to the increased TNF-α observed with low-dose estradiol exposures. Indeed, we found that higher levels of maternal TNF-α were associated with lower risk of adult depression in analyses of males and females combined, suggesting that some aspects of the neurodevelopmental effects of maternal HPA-immune activity do not vary between males and females. The sex difference in the association between maternal immune activity and offspring's risk of depression was because of sex-dependent variation in TNF-α *relative to* IL-10, and, therefore, we speculate the inability of the mother to mount an anti-inflammatory (IL-10) response to adverse stimuli. Thus, in the context of higher estradiol levels during pregnancy, a maternal TNF-α deficit in tandem with a higher anti-inflammatory response may have different consequences if the mother is carrying a female versus a male fetus.

There are other potential reasons for the association between lower TNF-α and offspring's risk of depression. Lower maternal TNF-α has been associated with a higher mean glycemic index during third trimester, implicating a potential role for metabolic pathways during pregnancy.^51^ Reduced maternal TNF-α levels could also result from dysregulation of the parasympathetic nervous system following an immune response, as abnormal patterns of autonomic arousal are implicated in sex-dependent risk for depression.^17^ Further, nicotinic cholinergic signals transmitted by the vagus nerve to the central nervous system after bacterial endotoxin exposure can inhibit TNF-α expression.^52^

It is likely that multiple mechanisms regulate the effects of maternal immune activity on offspring brain development, and that the specific mechanisms depend on the gestational timing of exposure. These include the following: dysregulation of nerve growth factors; loss of dendritic connections and white matter connectivity; apoptosis; dysregulation of neurotransmitters such as gamma aminobutryic acid and dopamine; and hormonal dysregulation.^48, 53, 54^ These mechanisms may individually and/or collectively impede the sexual differentiation of the brain during fetal development^22^ in the context of immune, infectious and/or psychosocial stressors. Future work is needed to provide evidence for one or more of these mechanisms.

Potential limitations of this study are important to consider. First, our findings are dependent on the validity of cytokine assays performed on prenatal sera drawn in the 1960s and stored at −20 °C. However, the stability of many types of analytes from stored CPP serum has been demonstrated.^25, 55^ Although artifacts introduced through the collection and freezing of the samples cannot be ruled out,^56^ all samples used in these analyses were treated comparably to one another with respect to the processing and freeze/thaw cycles, and all assays employed reagents obtained from the same lot and were conducted blind with respect to offspring's depression status. Population norms of cytokine concentrations in maternal sera during pregnancy are not generally available. Recent studies of maternal gestational sera in contemporary samples report highly varying concentrations.^57, 58^ However, relative between-group differences in cytokine levels, as demonstrated here with respect to offspring sex, should be considered robust when consistent assay methods and reagents are used across groups. To provide maximum comparability across assay runs, our laboratory additionally included a full set of serial cytokine standards in every assay along with quality-control samples and a constant in-house control sample to ensure that inter-assay covariance was minimized. Second, our use of two different assessment instruments to determine the presence of major depression is a potential source of measurement variance that would be expected to attenuate associations. However, these instruments have been shown to be highly concordant for the diagnosis of major depressive episode. In addition, we controlled for diagnostic instrument (SCID versus CIDI) in all analyses.^24^ Third, the CPP did not administer psychiatric interviews to participants during pregnancy, raising the possibility of confounding by maternal psychopathology. However, results were unchanged when we controlled for the presence of ‘psychosis or neurosis’ on the obstetric diagnostic summary (data not shown).

In summary, our results suggest that variations in maternal immune system activity during pregnancy (specifically TNF-α:IL-10) have significant sex-dependent effects on the development of major depression in adult offspring. Given these findings, an important direction for future research is to determine whether immune activation disturbances that have been observed in adult depression have fetal origins. Finally, the finding of a sex-dependent association between prenatal *maternal* immune responses and offspring’s risk for depression warrants further investigation of the developmental timing of prenatal challenges and the impact on HPA and HP-gonadal axes’ responses that influence stress-immune pathways.

## Figures and Tables

**Table 1:** Descriptive characteristics of the sample of depression cases (recurrent and lifetime) and non-depressed controls (*n*=1258)

	*Cases with major depression (*n=*484)*	*Controls without major depression (*n=*774)*
*Demographic characteristics of participants*		
Female sex, % (*N*)	66.7 (323)	54.1 (419)
White race/ethnicity, % (*N*)	90.5 (438)	91.0 (704)
Mean (s.d.) age at interview	39.7 (3.8)	39.6 (3.4)
Assessed with SCID, % (*N*)	45.9 (222)	34.1 (264)
Mean (s.d.) days gestation at sample collection	226.0 (22.1)	227.6 (24.6)
		
*Cytokine concentrations (pg ml^−1^) in maternal serum (median, interquartile range)*		
IL-1β	1.1 (5.0)	1.0 (6.3)
IL-6	1.1 (2.9)	1.2 (3.6)
IL-10	1.7 (2.5)	2.0 (2.6)
TNF-α	3.4 (2.5)	3.6 (2.7)
TNF-α:IL-10	1.8 (2.6)	1.7 (2.6)
		
*Female offspring (*n=*323 cases*, n=*419 controls)*		
IL-1β	1.1 (5.2)	1.0 (5.8)
IL-6	1.2 (3.1)	1.1 (3.3)
IL-10	1.8 (2.6)	2.0 (2.4)
TNF-α	3.4 (2.5)	3.5 (2.7)
TNF-α:IL-10	1.7 (2.5)	1.7 (2.7)
		
*Male offspring (*n=*161 cases*, n=*355 controls)*		
IL-1β	1.3 (3.5)	1.0 (7.0)
IL-6	1.1 (1.9)	1.2 (4.4)
IL-10	1.6 (2.1)	2.1 (3.2)
TNF-α	3.4 (2.6)	3.6 (2.7)
TNF-α:IL-10	2.0 (3.3)	1.7 (2.5)

Abbreviations: DSM-IV, Diagnostic and Statistical Manual, fourth edition; IL, interleukin; SCID, Structured Clinical Interview for DSM-IV; TNF, tumor necrosis factor.

**Table 2:** ORs for major depression associated with concentrations of maternal pro- and anti-inflammatory cytokines in late-second and early-third trimester maternal serum^a^

	*Association between cytokine quintiles and offspring risk of depression*					
	*<20th percentile OR (CI)*	*20–40th percentile OR (CI)*	*41–60th percentile OR (CI)*	*61–80th percentile OR (CI)*	*>80th percentile OR (CI)*	Sex*cytokine interaction test^*2*^ χ^*2*^*, df=4 (*P*)*
IL-1β	1	0.76 (0.53, 1.08)	1.18 (0.83, 1.68)	1.08 (0.76, 1.55)	0.78 (0.54, 1.14)	1.3 (0.862)
IL-6	1	0.90 (0.64, 1.27)	1.05 (0.74, 1.49)	0.87 (0.61, 1.24)	0.87 (0.60, 1.28)	1.3 (0.863)
TNF-α	1	0.80 (0.56, 1.16)	0.75 (0.52, 1.07)	0.85 (0.59, 1.22)	0.68 (0.48, 0.98)*	3.4 (0.498)
IL-10	1.17 (0.81, 1.70)	1.04 (0.72, 1.51)	0.94 (0.65, 1.37)	0.77 (0.53, 1.13)	1	7.4 (0.118)
TNF-α:IL-10	1	0.77 (0.54, 1.11)	0.77 (0.53, 1.12)	0.95 (0.66, 1.35)	0.82 (0.57, 1.18)	13.9 (0.008)*
						
*Sex-specific results*^b^						
TNF-α (females)	1	0.71 (0.45, 1.13)	0.84 (0.54, 1.31)	0.82 (0.52, 1.29)	0.69 (0.44, 1.08)	
TNF-α (males)	1	1.06 (0.57, 1.94)	0.63 (0.33, 1.19)	0.98 (0.54, 1.79)	0.73 (0.39, 1.35)	
IL-10 (females)	0.78 (0.49, 1.25)	0.81 (0.50, 1.30)	0.76 (0.47, 1.24)	0.67 (0.41, 1.08)	1	
IL-10 (males)	2.12 (1.14, 3.93)*	1.52 (0.83, 2.75)	1.30 (0.70, 2.39)	0.88 (0.45, 1.69)	1	
TNF-α:IL-10 (females)	1	0.54 (0.34, 0.86)*	0.59 (0.37, 0.95)*	0.84 (0.53, 1.33)	0.51 (0.32, 0.81)*	
TNF-α:IL-10 (males)	1	1.41 (0.75, 2.67)	1.27 (0.68, 2.38)	1.20 (0.64, 2.25)	1.86 (1.02, 3.39)*	

Abbreviations: CI, confidence interval; CIDI, Composite International Diagnostic Interview; df, degrees of freedom; DSM-IV, Diagnostic and Statistical Manual, fourth edition; IL, interleukin; OR, odds ratio; SCID, Structured Clinical Interview for DSM-IV; TNF, tumor necrosis factor.

*P<0.05.

a ORs obtained from logistic regression models, also controlling for race/ethnicity, age at adult interview, type of interview (SCID versus CIDI) and gestational age at prenatal serum collection.

b Tests for sex*cytokine interactions from logistic regression models with the covariates listed in (a), adding four interaction terms between sex and cytokine quintiles. Sex-specific ORs and confidence intervals obtained from the coefficients in these models.
