# Supplementary material for: Prenatal immune programming of the sex-dependent risk for major depression
Source: Transl Psychiatry. 2016 May 31;6(5):e822–. doi: 10.1038/tp.2016.91 (PMC5545649; doi:10.1038/tp.2016.91)
Supplement: Supplementary Tables [file tp201691x1.pdf]

Supplementary Information for Gilman et al., Prenatal Immune Programming of Sex-Dependent Risk for Major Depression

Table S1 Median cytokine concentrations (pg/mL) within each quintile of the 4 inflammatory cytokines

Table S2 Concentrations (pg/mL) of cytokines in maternal serum linked to offspring with recurrent depression (n=277)

Table S3 Odds ratios for *recurrent* major depression associated with concentrations of maternal pro- and anti-inflammatory cytokines in maternal serum

Table S4 Odds ratios for lifetime major depression associated with concentrations of maternal pro- and anti-inflammatory cytokines in maternal serum, removing from the analysis sample participants linked to prenatal serum samples drawn on the date of delivery

Table S1. Median cytokine concentrations (pg/mL) within each quintile of the 4 inflammatory cytokines

	**<20th percentile**	**20-40th percentile**	**41-60th percentile**	**61-80th percentile**	**>80th percentile**
IL-1β	0.055	0.271	1.161	4.424	18.747
IL-6	0.150	0.577	1.259	3.136	18.262
TNF-α	1.384	2.617	3.540	4.680	7.284
IL-10	0.462	1.175	1.964	3.162	10.965

Table S2. Concentrations (pg/mL) of cytokines in maternal serum linked to offspring with recurrent depression (n=277).

	**Median (interquartile range)**
Both sexes (n=277 cases)	
IL-1β	1.1 (5.0)
IL-6	1.1 (2.8)
IL-10	1.6 (2.4)
TNF-α	3.1 (2.4)
TNF-α:IL-10	1.8 (2.6)
Female offspring (n=196 cases)	
IL-1β	1.1 (5.9)
IL-6	1.2 (3.0)
IL-10	1.6 (2.4)
TNF-α	3.1 (2.4)
TNF-α:IL-10	1.7 (2.6)
Male offspring (n=81 cases)	
IL-1β	1.1 (3.5)
IL-6	1.0 (1.9)
IL-10	1.6 (2.5)
TNF-α	3.3 (2.6)
TNF-α:IL-10	2.0 (3.0)

Table S3. Odds ratios for *recurrent* major depression associated with concentrations of maternal pro- and anti-inflammatory cytokines in maternal serum[1]

	Association between cytokine quintiles and offspring risk of recurrent depression					
	**<20th percentile OR (CI)**	**20-40th percentile OR (CI)**	**41-60th percentile OR (CI)**	**61-80th percentile OR (CI)**	**>80th percentile OR (CI)**	**Sex*cytokine interaction test[2] χ^2, df=4 (P)**
TNF-α	1	0.78 (0.52, 1.18)	0.62 (0.41, 0.95)*	0.63 (0.40, 0.96)*	0.51 (0.33, 0.80)*	5.3 (0.258)
IL-10	1.34 (0.84, 2.12)	1.25 (0.80, 1.95)	0.99 (0.62, 1.58)	0.78 (0.49, 1.26)	1	2.6 (0.627)
TNF-α:IL-10	1	0.78 (0.50, 1.22)	0.73 (0.46, 1.14)	1.01 (0.66, 1.54)	0.77 (0.49, 1.19)	8.8 (0.067)
TNF-α (Females)	1	0.72 (0.44, 1.20)	0.72 (0.44, 1.19)	0.53 (0.31, 0.92)	0.50 (0.29, 0.86)	
TNF-α (Males)	1	1.03 (0.49, 2.14)	0.44 (0.19, 1.04)	0.93 (0.44, 1.97)	0.63 (0.28, 1.39)	
IL-10 (Females)	1.00 (0.57, 1.76)	1.02 (0.58, 1.79)	0.82 (0.46, 1.47)	0.72 (0.40, 1.29)	1	
IL-10 (Males)	1.89 (0.85, 4.19)	1.62 (0.76, 3.45)	1.32 (0.61, 2.89)	0.75 (0.31, 1.81)	1	
TNF-α:IL-10 (Females)	1	0.55 (0.32, 0.94)*	0.53 (0.31, 0.94)*	0.89 (0.53, 1.49)	0.51 (0.29, 0.88)*	
TNF-α:IL-10 (Males)	1	1.54 (0.67, 3.54)	1.42 (0.63, 3.22)	1.38 (0.61, 3.09)	1.73 (0.78, 3.86)	

*P<0.05

[1]Odds ratios obtained from logistic regression models, also controlling for race/ethnicity, age at adult interview, type of interview (SCID vs. CIDI), and gestational age at prenatal serum collection. Models estimated among 277 cases of recurrent depression, and 774 controls without depression.

[2]Tests for sex*cytokine interactions from logistic regression models with the covariates listed in (a), adding 4 interaction terms between sex and cytokine quintiles. Sex-specific odds ratios and confidence intervals obtained from the coefficients in these models.

Table S4. Odds ratios for lifetime major depression associated with concentrations of maternal pro- and anti-inflammatory cytokines in maternal serum, removing from the analysis sample participants linked to prenatal serum samples drawn on the date of delivery[1]

	**Association between cytokine quintiles and offspring risk of depression**					
	**<20th percentile OR (CI)**	**20-40th percentile OR (CI)**	**41-60th percentile OR (CI)**	**61-80th percentile OR (CI)**	**>80th percentile (Reference) OR (CI)**	**Test for sex * cytokine interaction[2] χ^2, df=4 (P)**
TNF-α	1	0.75 (0.51, 1.09)	0.72 (0.50, 1.05)	0.75 (0.51, 1.10)	0.66 (0.45, 0.97)*	3.6 (0.462)
IL-10	0.97 (0.65, 1.46)	0.85 (0.57, 1.27)	0.77 (0.51, 1.17)	0.65 (0.43, 0.98)*	1	7.8 (0.101)
TNF-α:IL-10	1	0.68 (0.45, 1.01)	0.63 (0.42, 0.94)*	0.77 (0.52, 1.14)	0.71 (0.48, 1.05)	10.4 (0.034)*
Sex-specific estimates[2]						
TNF-α (Females)	1	0.67 (0.41, 1.08)	0.83 (0.52, 1.30)	0.71 (0.45, 1.15)	0.70 (0.44, 1.13)	
TNF-α (Males)	1	0.97 (0.52, 1.80)	0.20 (0.30, 1.13)	0.28 (0.46, 1.63)	0.65 (0.34, 1.25)	
IL-10 (Females)	0.63 (0.37, 1.05)	0.61 (0.36. 1.04)	0.59 (0.35, 1.01)	0.54 (0.32, 0.92)*	1	
IL-10 (Males)	1.93 (0.97, 3.84)	1.41 (0.72, 2.79)	1.19 (0.60, 2.39)	0.82 (0.39, 1.70)	1	
TNF-α/IL10 (Females)	1	0.53 (0.32, 0.87)*	0.51 (0.31, 0.86)*	0.72 (0.44, 1.19)	0.47 (0.28, 0.77)*	
TNF-α/IL10 (Males)	1	1.16 (0.57, 2.35)	1.04 (0.52, 2.10)	1.01 (0.50, 2.03)	1.61 (0.83, 3.14)	

*P<0.05

[1]Odds ratios obtained from logistic regression models, also controlling for race/ethnicity, age at adult interview, type of interview (SCID vs. CIDI), and gestational age at prenatal serum collection. Models estimated among 452 cases of depression and 692 controls without depression.

[2]Tests for sex*cytokine interactions from logistic regression models with the covariates listed in (a), adding 4 interaction terms between sex and cytokine quintiles. Sex-specific odds ratios and confidence intervals obtained from the coefficients in these models.
